# Perinatal Exposure to Low-Dose Methoxychlor Impairs Testicular Development in C57BL/6 Mice

**DOI:** 10.1371/journal.pone.0103016

**Published:** 2014-07-21

**Authors:** Xiaohong Du, Hua Zhang, Yuanwu Liu, Wanpeng Yu, Chaobin Huang, Xiangdong Li

**Affiliations:** State Key Laboratory of AgroBiotechnology, Faculty of Biological Sciences, China Agricultural University, Beijing, China; University Hospital of Münster, Germany

## Abstract

Methoxychlor (MXC), an organochlorine pesticide, has adverse effects on male reproduction at toxicological doses. Humans and wild animals are exposed to MXC mostly through contaminated dietary intake. Higher concentrations of MXC have been found in human milk, raising the demand for the risk assessment of offspring after maternal exposure to low doses of MXC. In this study, pregnant mice (F0) were given intraperitoneal daily evening injections of 1 mg/kg/d MXC during their gestational (embryonic day 0.5, E0.5) and lactational periods (postnatal day 21.5, P21.5), and the F1 males were assessed. F1 testes were collected at P0.5, P21.5 and P45.5. Maternal exposure to MXC disturbed the testicular development. Serum testosterone levels decreased, whereas estradiol levels increased. To understand the molecular mechanisms of exposure to MXC in male reproduction, the F1 testes were examined for changes in the expression of steroidogenesis- and spermatogenesis- related genes. RT-PCR analysis demonstrated that MXC significantly decreased *Cyp11a1* and increased *Cyp19a1*; furthermore, it downregulated certain spermatogenic genes (*Dazl*, *Boll*, *Rarg*, *Stra8* and *Cyclin-a1*). In summary, perinatal exposure to low-dose MXC disturbs the testicular development in mice. This animal study of exposure to low-dose MXC in F1 males suggests similar dysfunctional effects on male reproduction in humans.

## Introduction

Endocrine-disrupting compounds (EDCs) are synthetic or natural compounds that interfere with endogenous endocrine actions [1]. Studies have shown that EDCs, which include common pesticides such as methoxychlor, can influence sex differentiation [2], [3], [4], semen quality [5], [6], [7] and steroid production [8], [9], [10], [11].

Methoxychlor [1,1,1-trichloro-2,2-bis(4-methoxyphenyl) ethane; MXC] is a chlorinated hydrocarbon pesticide in common, worldwide used as a replacement for DDT [1,1,1-trichloro-2,2-bis(p-chlorophenyl)ethane]. MXC and its major metabolites, mono-desmethyl-MXC (OH-MXC) and bisdesmethyl-MXC (HPTE), possess ERα agonist, ERβ antagonist and antiandrogenic activities [12], [13], [14]. Perinatal exposure to high-dose MXC is able to cause many reproductive abnormalities in adult male mice or rat [15], [16], [17], [18], [19], [20], [21]. Amstislavsky et al. reported that transient exposure to 166.7 mg/kg/d MXC at embryonic day (E) 2 to E4 significantly increased the seminal vesicles weight and decreased the testosterone (T) level in male mice at 6 months [15]. Perinatal and juvenile oral treatment of rats with 150 mg/kg/d or 60 mg/kg/d MXC reduced testicular size, decreased the number of spermatogonia and Sertoli cells and serum LH and FSH in those animals at adults [16], [18]. Cupp et al. demonstrated that transient exposure to 150 mg/kg/d MXC from E7 through E15 reduced the number of germ cells in rat at postnatal-day (P) 17-P20 and increased the number of apoptotic elongating spermatids in rat at P60 [17]. Most previous studies were carried out using toxicological doses of MXC (over 100 mg/kg/d) [22], [23], even though it is logically implausible that humans or wild life would be exposed under such conditions. Research on the effects on the male reproductive system of low doses or environmentally relevant doses of MXC (less than 2 mg/kg/d) [24], [25] is urgently required. Clinically, impaired reproductive development has been demonstrated in the sons of female gardeners or farmers who have been exposed to pesticides [26], [27], [28], [29], [30], [31]. Humans and wild animals are exposed to MXC mostly through the dietary intake of contaminated food. Higher concentrations of MXC have been found in human milk, elevating the demand for assessing the risk of offspring after maternal exposure to MXC. To clarify the molecular mechanisms of exposure to MXC on male reproduction, we conducted a study examining MXC exposure during the perinatal period to determine its effect on fetal development, as well as the long-term impacts on the male reproductive systems of C57BL/6 mice.

## Materials and Methods

### Ethics Statement

The Ethics Committee for Animal Experimentation of the China Agricultural University approved all of the animal experiments (Register No. SKLAB-2011-01-05). All surgeries were performed using sodium pentobarbital anesthesia, and efforts were made to minimize animal suffering.

### Experimental design: *In vivo* mouse studies

Female C57BL/6 mice were purchased from Weitonglihua, Beijing (under the license of Charles River, US). To test the appropriate perinatal exposure dose of MXC, a pilot study was carried out on gestating C57BL/6 mice (F0 generation). The female mice were given intraperitoneal daily (at 20∶00) injections of MXC (0.25 µg/kg/d, 2.5 µg/kg/d, 20 µg/kg/d, 1 mg/kg/d) from plug date (E0.5) to weaning (P21.5), and the male offspring (F1) were assessed (n = 6–8/group). The control group received the vehicle alone (sesame oil). To determine the effects of different ways of MXC exposure, intraperitoneal injection or intragastrical administration of 1 mg/kg/d MXC were studied. There was no significant difference between the intraperitonally injected and intragastrically administrated plasma or adipose tissue MXC concentrations (Fig. S1). Thus for further experiments we have used intraperitoneal MXC injections. The concentrations of MXC in plasma and adipose tissue in female mice are comparable to the concentrations in women (2.23 ng/ml and 24.38 ng/g in mice, 0.38 ng/ml and 29.86 ng/g in women) [32]. Moreover, as we obtained significant results from the 1 mg/kg/d MXC-treated group, the later analyses were conducted in this group. The majority of animals were tested by P45.5 of age. The testes from control and MXC F1 generation mice were collected at three time points (P0.5, P21.5 and P45.5), and the body and testes weights were recorded simultaneously.

### Intraperitoneal injection and intragastrical administration of MXC

To determine the effects of different ways of MXC exposure, female C57BL/6 mice were given daily (at 20∶00) intraperitoneal injections or intragastrical administration of 1 mg/kg/d MXC for 5 days (n = 4/group). Then the mice were anesthetized, and blood and abdominal adipose tissue were colleted for MXC detection.

### Detection of MXC with ELISA

Concentration of MXC in the plasma and adipose tissue were measured by ELISA following the manufacturer’s protocol (R&D Systems, US). Briefly, adipose tissue was homogenated and extracted with 70% Methanol (2 ml/g adipose tissue), then filtered with Whatman No 1. filter paper. After proper dilution, standards and samples were added to a 96-well plate (Coated with MXC coupling antigen). The antibody-enzyme conjugate was then added, and the plate was incubated for 30 min at 37°C and washed. Next the color-reagents A and B were added to the well and incubated for 10 min at 37°C. The stop solution was then added and the absorbance value at 450 nm was read. In order to calculate the sample’s concentration, the standard curve was generated according to the standards.

### Sperm count and motility

Epididymis sperm was obtained as previously described [33]. Caudae epididymidis and vasa deferentia were excised and then rinsed with medium containing 150 mM NaCl, 5 mM KCl, 2 mM CaCl_2_, 1 mM MgCl_2_, 30 mM HEPES, 10 mM glucose, 10 mM lactic acid, and 1 mM pyruvic acid (pH 7.4). After transfer to 1 ml of medium supplemented with 5 mg of bovine serum albumin per ml and 15 mM NaHCO_3_, semen was allowed to exude (15 min at 37°C, 5% CO_2_) from three to five small incisions. Total sperm number was determined by using a Neubauer hemocytometer as previously described [34]. The percentage of motile sperms was recorded using a phase contrast microscope at a magnification of 400×. To determine sperm motility, 100 sperm each were observed in 3 different fields, and classified into motile and non-motile sperms, and the motility was expressed as percentage incidence.

### Histology analyses and quanlification of germ and Leydig cell number

Testes were fixed in 10% neutral buffered formalin or Bouin’s (Sigma-Aldrich, US), embedded in paraffin, sectioned in 5 µm, and then stained with hematoxylin and eosin (H&E), according to standard procedures. Quantification of the number of germ cells at different stages was carried out as described previously (n = 8/group) [35]. Briefly, the nuclei of different germ cells were counted in 100 round seminiferous tubules cross sections, chosen at random for each mouse. The counts were subjected to Abercrombie correction for section thickness and differences in the nucleus or nucleolar diameter [36], as modified by Amann [37]: average number of cells = [number of nuclei counted×thickness (µm) of section]/[average diameter of nuclei (µm)+thickness (µm) of section]. The number of Leydig cells was measured, as described previously (n = 7/group) [38].

### Measurement of plasma sex hormones

T was measured by radioimmunoassay after diethyl ether extraction, as described previously (n = 4–5/group) [39]. Concentrations of estradiol (E_2_) were measured using a commercial radioimmunoassay kit (Immunotech, France) (n = 4–5/group).

### RNA extraction, RT-PCR and semi-quantitative RT-PCR

Total RNA was extracted from frozen testicular tissues or cells using the acid guanidinium method [40] and stored at −80°C until assayed. RT-PCR was performed as previously [41], with one microgram of total RNA being incubated with 10 IU avian myeloblastosis virus reverse transcriptase (Promega, US) at 37°C for 1 h. The cDNAs were then denatured at 95°C for 5 min and amplified through 25 cycles of PCR, using the following conditions: 94°C for 30 sec, 56°C to 61°C for 45 sec, and 72°C for 45 sec. An aliquot of the RT-PCR product was subjected to agarose gel electrophoresis and visualized by ethidium bromide staining (n = 6/group). Each sample was run in triplicate, and three individual experiments were conducted. Ribosomal protein L19 gene was amplified as an internal control. The primers used for the various RT-PCR analyses were given in Table 1. The gel bands were quantified using ImageJ software version 1.42 (Image processing and analysis in Java, NIH, Bethesda, MD; http://rsb.info.nih.gov/ij/download.html).

**Table 1:** List of primer pairs.

Gene	Forward primer	Reverse primer
*L19*	5′-gaaatcgccaatgccaact-3′	5′-tgagactcgcaggtctaaga-3′
*Insl3*	5′-cgctgctactactgatgctcc-3′	5′-caggtcttgctgggtgc-3′
*Hsd3b*	5′-aatctgaaaggtacccagaa-3′	5′- tcatcatagctttggtgagg-3′
*Cyp11a1*	5′-gctgcctgggatgtgtgattt-3′	5′-cggaagtgggtggtatttt-3′
*Star*	5′-cgggtggatgggtcaagttc-3′	5′-ccaagcgaaacaccttgcc-3′
*Nr5a1*	5′-tgcagaatggccgaccag-3′	5′-tggcggtagatgtggtc-3′
*Ar*	5′-ctgggaagggtctaccac-3′	5′-ggtgctatgttagcggcctc-3′
*Esr1*	5′-cgtgtgcaatgactatgcctc-3′	5′-tttcatcatgcccacttcgtaa-3′
*Esr2*	5′-ctgtgcctcttctcacaagga-3′	5′-tgctccaagggtaggatggac-3′
*Cyp19a1*	5′-atgaacgatccgtcaaggac-3′	5′-actcgagcctctgcattctt-3′
*Pcna*	5′-caacttggaatcccagaac-3′	5′-agacagtggagtggctttt-3′
*Sohlh2*	5′-tgctctgaactgctgaagaa-3′	5′-gagctctcggaaacagagcc-3′
*Dazl*	5′-atgtctgccacaacttctgag-3′	5′-ctgatttcggtttcatccatcct-3′
*Boll*	5′-gggattcctcgttctagtctca-3′	5′-gaaggccaagatggtggga-3′
*Rarg*	5′-ggagcaggcttcccattcg-3′	5′-catggcttatagacccgagga-3′
*Stra8*	5′-gtttcctgcgtgttccacaag-3′	5′-cacccgaggctcaagcttc-3′
*Cyclin-d2*	5′-cgatgattgcaactggaagc-3′	5′-ttcagcagcagagcttcgat-3′
*Nanos3*	5′-tgcaggcaaaaagctgacc-3′	5′-cttcctgccacttttggaac-3′
*Pou5f1*	5′-aagttggagaaggtggaacc-3′	5′-tgatcctcttctgcttcagc-3′
*Cyclin-a1*	5′-gatgtgtatgaagtcgacacc-3′	5′-gtggggtcaaccagcattgg-3′

### Western blotting

The total protein was extracted in the extraction buffer [20% glycerol, 50 mmol/L Tris-HCl (pH 6.8) and 0.5% (v/v) Tween 20], to which a protease inhibitor cocktail (Sigma-Aldrich, US) was added. Next, the samples were centrifuged at 12,000 rpm (6439 g) for 10 min to remove nondissolved material. The protein concentration in the supernatant was measured using a Tiangen protein assay kit (Tiangen Biotech, China). Aliquots of protein extract containing 30 µg of total protein were electrophoresed in a 10% SDS-PAGE (sodium dodecyl sulfate-polyacrylamide gel electrophoresis) and transferred to a P-membrane (Millipore, US) for 3 h with transfer buffer (25 mmol/L Tris, 192 mmol/L glycine, and 20% (v/v) methanol). The membrane was blocked with 2% dried nonfat milk and incubated with antibodies against L19 (sc-100830, Santa Cruz, US, 1∶500) and Stra8 (ab49602 Abcam, US, 1∶500). After treatment with the primary antibody, the membrane was washed in Tris-buffered saline-Tween buffer (20 mmol/L Tris, 500 mmol/L NaCl, 0.05% Tween 20) and incubated with the secondary antibody (dilution at 1∶3000). Final exposure was achieved using enzymatic chemiluminescence (Amersham Pharmacia, UK). The films were scanned and quantified using ImageJ software (n = 6/group).

### Cell culture and MXC exposure

Murine Leydig tumor cell line (mLTC-1, ATCC CRL-2065, US) and germ cell line GC-1 spg (ATCC CRL-2053, US) were purchased from ATCC. mLTC-1 cells were maintained in RPMI-1640 medium (Sigma-Aldrich, US) and GC-1 cells were maintained in Dulbecco’s modified Eagle medium nutrient mixture F-12 medium (DMEM/F-12, Sigma-Aldrich, US), media were supplemented with 10% fetal bovine serum (Sigma-Aldrich, US). The cells were cultured at 37°C in a humidified atmosphere of 5% CO_2_. MXC (Sigma-Aldrich, US) was dissolved in dimethyl sulfoxide (DMSO, Sigma-Aldrich, US) to 0.1 M and diluted with media to 10^−6^ M. DMSO at 0.05% in serum-free media was used as a vehicle control. All of the experiments were incubated for 24 h (n = 4/group).

### *In vitro* testicular cultures and treatments

The testes were dissected from new born C57BL/6 mice in PBS solution. Testes were cultured atop 1.5- to 1.7-ml agar blocks, as described [42]. All-trans Retinoic acid (RA, Sigma-Aldrich, US) was dissolved in ethanol, and Busulfan (Sigma-Aldrich, US) and MXC (Sigma-Aldrich, US) were dissolved in DMSO. For busulfan treatment, busulfan (40 mg/kg body weight) was injected intraperitoneally into pregnant female mice at 9.5 days *post coitum* [43]. The concentrations of the compounds in the culture media were as follows: all-trans RA, 0.7 M; and MXC, 10^−6 ^M. Control cultures were treated with ethanol and/or DMSO, as appropriate.

### *In situ* hybridization

For *in situ* hybridization, the testes were fixed in 4% paraformaldehyde at 4°C overnight. Whole-mount *in situ* hybridization was performed as reported [44]. The digoxigenin-labeled RNA probe for *Stra8* was detected using an alkaline phosphatase-conjugated antidigoxigenin antibody and through staining with a BM purple alkaline phosphatase substrate (Roche, US) (n = 4/group). RA stimulates the expression of *Stra8* [45] and the RA-treated testes served as a positive control. Busulfan could mediate the depletion of germ cell [46] and the busulfan-treated testes served as a negative control.

### Statistical analysis

The data were analyzed for statistical significance with the SPSS 12.0.1 Package (SPSS Inc., US). Data for all groups were first tested for normality with the Shapiro-Wilk test. If the group’s data were normally distributed, they were compared using a one-way analysis of variance. *P* values <0.05 were regarded as statistically significant. All values are presented as the means ± SEM (standard error of mean). All of the graphs were generated with GraphPad Prism 5.0 (GraphPad Software Inc., US).

## Results

### MXC affects both the body and testicular weights of F1 generation male mice

Gestating C57BL/6 mice (F0 generation) were given MXC (0.25 µg/kg/d, 2.5 µg/kg/d, 20 µg/kg/d or 1 mg/kg/d) from E0.5 of gestation to P21.5 of weaning. Both the body and testicular weights of F1 males were recorded at the three time points (P0.5, P21.5 and P45.5). At P0.5, the exposure of pregnant mice to 20 µg/kg/d and 1 mg/kg/d MXC significantly decreased the neonatal weights of pups (Table 2). At P21.5, the body weights of all MXC-treated groups were not affected, compared with control group; however, the absolute and relative testicular weights of all MXC-treated groups increased significantly (Table 2, 3). At P45.5, the body weights of the MXC-treated groups increased significantly compared to the control group, but the absolute testicular weights were not altered and the relative testicular weights were decreased significantly (Table 2, 3).

**Table 2:** Effects of MXC on body weights and testes weights.

Treatment group	B.W.(g) P0.5	testis weight(g)/B.W.(g) P21.5	testis weight(g)/B.W.(g) P45.5
Control	1.58±0.14	0.0285±0.0078/7.95±0.36	0.0763±0.0082/17.91±1.41
MXC(250 ng/kg/d)	1.53±0.14	0.0347±0.0030*/8.09±0.60	0.0704±0.0068/20.52±0.80*
MXC(2.5 µg/kg/d)	1.54±0.14	0.0324±0.0061*/8.19±1.02	0.0690±0.0084/21.33±1.22*
MXC(20 µg/kg/d)	1.39±0.11*	0.0311±0.0039*/7.93±0.51	0.0679±0.0017/20.36±1.61*
MXC(1 mg/kg/d)	1.46±0.12*	0.0335±0.0041*/8.14±1.34	0.0682±0.0012/20.97±1.72*

Table 2 shows the body weights and absolute testes weights, as compared between control F1 mice and treatment groups at postnatal day 0.5 (P0.5), P21.5 and P45.5. At least 20 F1 male mice per group were enrolled for statistical analysis. *indicates *P*<0.05.

**Table 3:** Effects of MXC on relative testicular weights.

Treatment group	testis weight(mg/g B.W.) P21.5	testis weight(mg/g B.W.) P45.5
Control	3.59±0.25	4.27±0.37
MXC(0.25 µg/kg/d)	4.28±0.13*	3.44±0.19*
MXC(2.5 µg/kg/d)	3.97±0.31*	3.25±0.52*
MXC(20 µg/kg/d)	3.91±0.28*	3.36±0.11*
MXC(1 mg/kg/d)	4.13±0.49*	3.27±0.23*

Table 3 shows the relative testes weights, as compared between control F1 mice and treatment groups at P21.5 and P45.5. At least 20 F1 male mice were enrolled for statistical analysis. *indicates *P*<0.05.

### MXC alters the testicular histology and cell numbers of F1 generation mice

**Figure 1:**
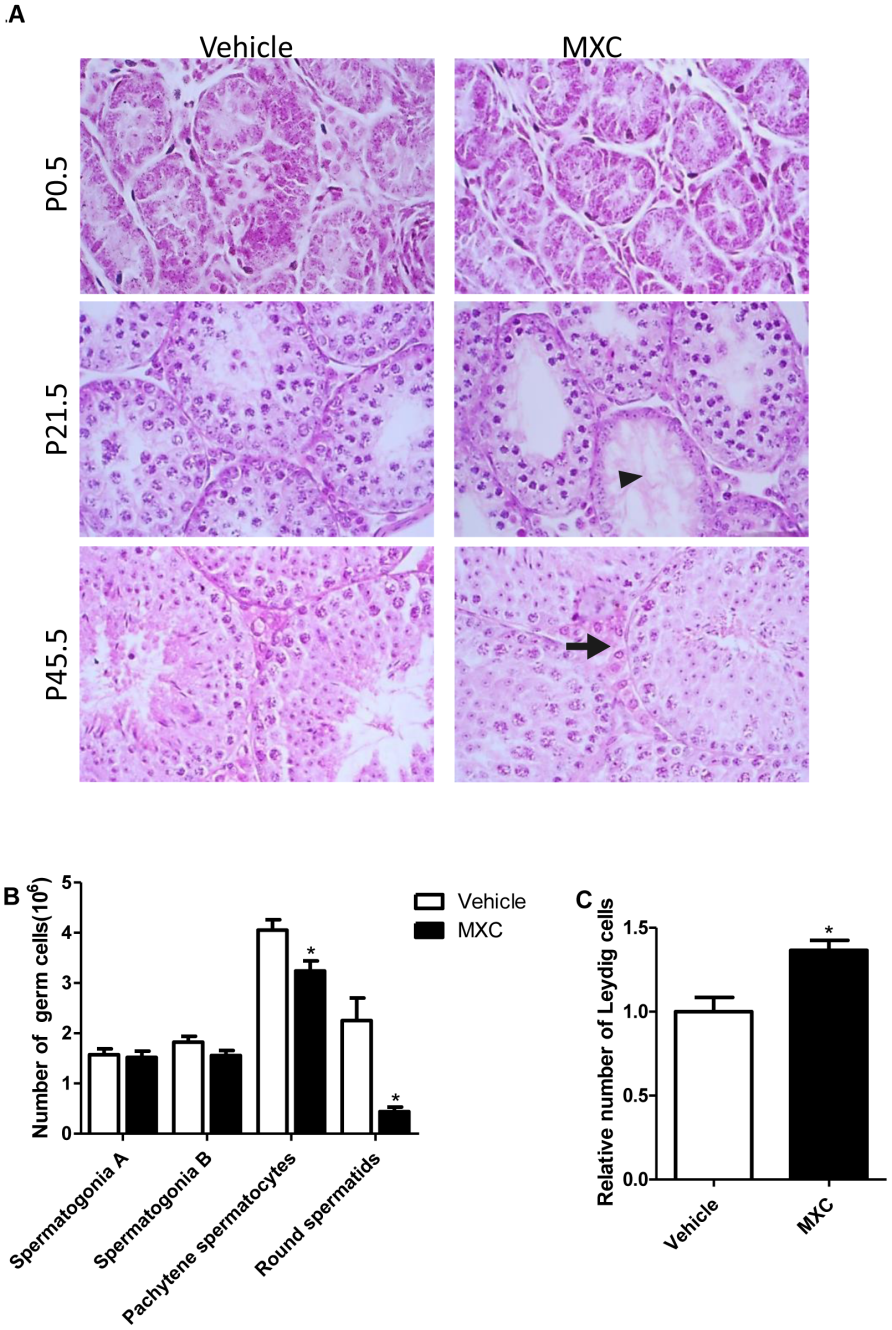
MXC alters the testicular histology and cell number of F1 generation mice. (**A**) Representative micrographs of H&E-stained testes of mice exposed to vehicle or 1 mg/kg/d MXC (n = 6–8/group). The arrow indicates tubules with less differentiated germ cells; the arrowhead indicates enlarged interstitial areas. Original magnification, ×100. (**B**) The quantification of the number of A spermatogonia, B spermatogonia, pachytene spermatocyte and round spermatid for P21.5 testes (n = 8/group). (**C**) The quantification of Leydig cells for P45.5 testes (n = 7/group). Vehicle-treated group was set at 1.0. The data represent the mean ± SEM. *P<0.05 versus vehicle.

The image quantification analysis demonstrated that the numbers of pachytene spermatocyte and round spermatid were significantly decreased at P21.5 in the 1 mg/kg/d MXC-treated group, compared with vehicle-treated animals (Fig. 1A, B). And the number of Leydig cells at P45.5 in the 1 mg/kg/d MXC-treated group was increased significantly (Fig. 1A, C). No histomorphology abnormality was observed in other MXC dosage groups (Fig. S2). However, prenatal exposure to 1 mg/kg/d MXC did not significantly affect the epididymal sperm count and motility at P45.5 (Fig. S3).

### MXC inhibits steroidogenesis in F1 generation mice testes

**Figure 2:**
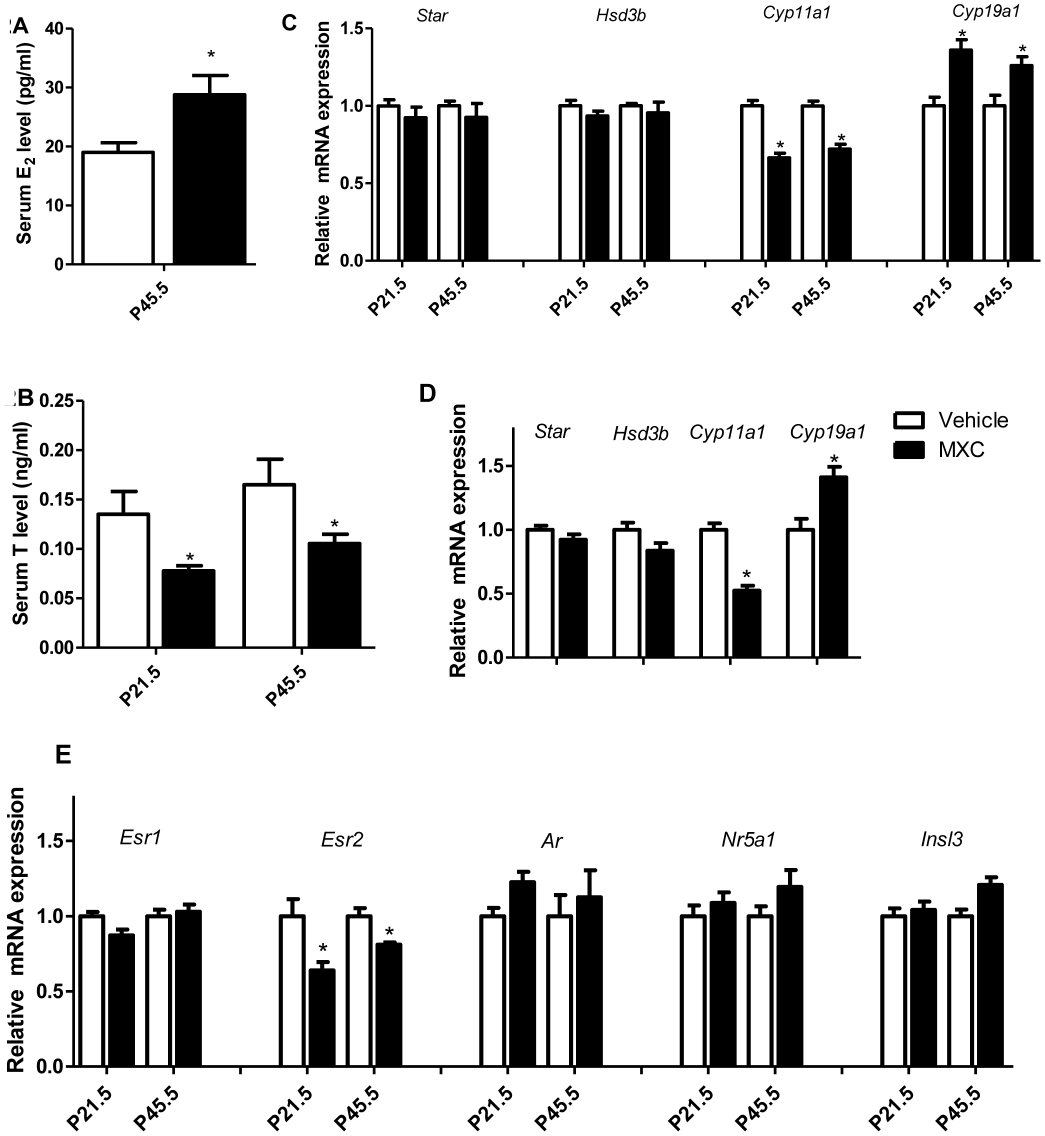
MXC inhibits steroidogenesis in F1 generation mice testes. (**A**, **B**) Relative plasma T and E_2_ levels in P21.5 and P45.5 mice exposed to vehicle or 1 mg/kg/d MXC (n = 4–5/group). (**C**) Testicular mRNA expression of the steroidogenic genes (*Star*, *Hsd3b*, *Cyp11a1* and *Cyp19a1*), normalized to *L19* level, in P21.5 and P45.5 testes of mice exposed to vehicle or 1 mg/kg/d MXC (n = 6 group). (**D**) The related mRNA expression of steroidogenic genes (*Star*, *Hsd3b*, *Cyp11a1* and *Cyp19a1*) was normalized to *L19* level in mLTC-1 cultured with DMSO or 10^−6^ M MXC for 24 h (n = 4/group). (**E**) Testicular mRNA expression of *Esr1*, *Esr2*, *Ar*, *Nr5a1* and *Insl3*, normalized to *L19* level, in P21.5 and P45.5 testes of mice exposed to vehicle or 1 mg/kg/d MXC (n = 6/group). Vehicle-treated group was set at 1.0. The data represent the mean ± SEM. *P<0.05 versus vehicle.

We observed a decrease in serum T and an increase in serum E_2_ concentrations in MXC-treated males (Fig. 2A, B). Also the steroidogenic genes were analyzed. We observed no changes in the mRNA levels of *steroidogenic acute regulatory protein* (*Star*) or *3 beta-hydroxysteroid dehydrogenase* (*Hsd3b*), while *cytochrome P450, family 11, subfamily a,* and *polypeptide 1* (*Cyp11a1*) was significantly decreased and *Cytochrome P450, family 19, subfamily a,* and *polypeptide 1* (*Cyp19a1*) was significantly increased (Fig. 2C). Moreover, we achieved similar results in MXC-treated mLTC-1 Leydig cells (Fig. 2D). We also analyzed the mRNA expressions of hormone nuclear receptors. We did not observe any significant change in the mRNA expression of *estrogen receptor α* (*Esr1*), *nuclear receptor subfamily 5, group A, member 1* (*Nr5a1*), *Androgen receptor* (*Ar*) or *Insulin-like 3* (*Insl3*), whereas *estrogen receptor β* (*Esr2*) mRNA expression was significantly decreased (Fig. 2E). We also re-confirmed these testicular mRNA expression results in MXC-treated mLTC-1 Leydig cell line that showed very similar trend towards the *in vivo* data (Fig. S4).

### MXC disturbs the meiosis in germ cells

**Figure 3:**
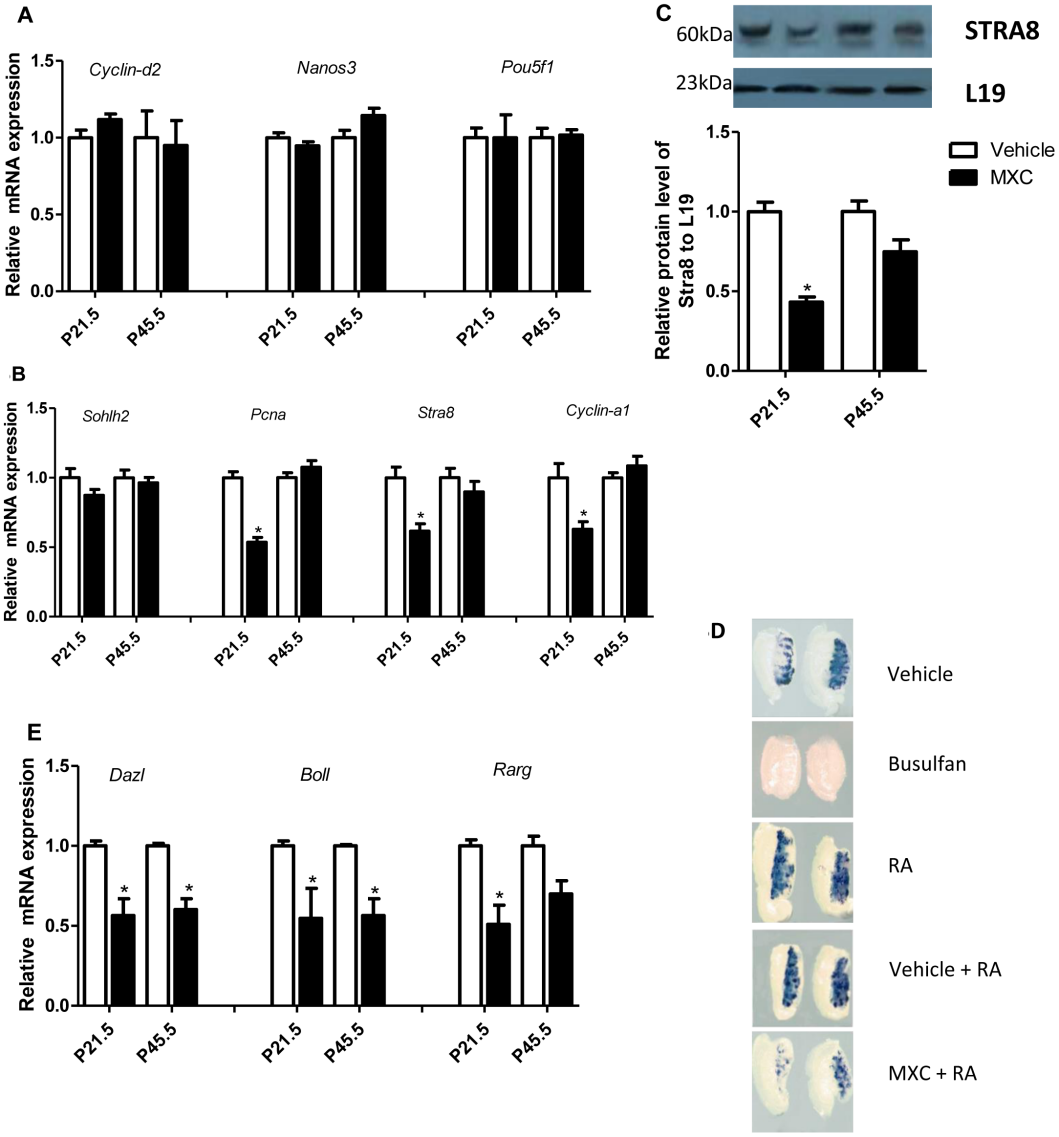
MXC disturbs the meiosis in germ cells. Related mRNA expression of *Cyclin-d2*, *Nanos3 and Pou5f1* (**A**), *Sohlh2*, *Pcna*, *Stra8* and *Cyclin-a1* (**B**) in P21.5 and P45.5 testes exposed to vehicle or 1 mg/kg/d MXC (n = 6/group), as analyzed by semi-quantitative RT-PCR. (**C**) Related protein levels of STRA8 in P21.5 and P45.5 testes of mice exposed to the vehicle or 1 mg/kg/d MXC (n = 6/group), as analyzed by western blotting. (**D**) Representative micrographs of *Stra8* expression in the testes treated with vehicle, busulfan, RA, Vehicle+RA, or RA+MXC for 48 h (n = 4/group), as analyzed by *in situ* hybridization. (**E**) Related mRNA expression of *Dazl*, *Boll* and *Rarg* in P21.5 and P45.5 testes of mice exposed to vehicle or 1 mg/kg/d MXC (n = 6/group), as analyzed by semi-quantitative RT-PCR. Relative expression levels were normalized to L19 level. Vehicle-treated group was set at 1.0. The data represent mean ± SEM. *P<0.05 versus vehicle.

At P21.5, the testes showed decreased numbers of pachytene spermatocyte and round spermatid in the 1 mg/kg/d MXC group (Fig. 1A, B). To determine the impact of MXC exposure on germ cell differentiation, we analyzed the expression of the undifferentiated spermatogonia specific-genes (*Cyclin-d2*, *Nanos3 and Pou5f1*), and no alteration was observed (Fig. 3A). We then checked the premeiotic germ cells marker in the testes and observed no change in the expression of *Sohlh2* mRNA in MXC-treated testes (Fig. 3B). However, the expressions of the proliferation marker *proliferating cell nuclear antigen* (*Pcna*), *stimulated by retinoic acid gene 8* (*Stra8*) and *Cyclin-a1* mRNA*s* were significantly decreased in the MXC-treated testes (Fig. 3B). As Stra8 plays a critical role in meiosis, we also checked the protein expression in the testes. The protein level of Stra8 was decreased significantly (Fig. 3C). Moreover, we confirmed the effect of MXC on *Stra8* with *in vitro* testicular culture and *in situ* hybridization. After treatment of 10^−6^ MXC for 48 h, the cultured testes revealed decreased *Stra8* expression compared to control group (Fig. 3D). In addition, the *Deleted in Azoospermia* gene family (*Dazl* and *Boll*) and *retinoic acid receptor gamma* (*Rarg*) mRNA expressions were significantly decreased in the MXC-treated testes (Fig. 3E). We also re-confirmed these testicular mRNA expression results in MXC-treated GC-1 cell line that showed very similar trend towards the *in vivo* data (Fig. S5).

## Discussion

Although, it is widely accepted that EDCs have a great influence on human and animal reproduction at toxicological doses, the knowledge on the exposure of humans to lower or environmentally relevant doses of EDCs or of related effects on offspring remains limited. The present study was carried out to investigate the mechanisms responsible for the adverse effects of low-dose MXC exposure during the perinatal period, allowing for a better understanding of the mechanisms behind these histological abnormalities in the prepubertal period. Our results demonstrated that perinatal exposure to low-dose MXC could impair the testes development, not only by disturbing testicular Leydig cell proliferation but also by interrupting steroidogenesis and spermatogenesis.

Previous experiments have demonstrated that exposure to organochlorine pesticides might induce intrauterine growth retardation (IUGR) [38], [39] and obesity in puberty [47], [48], [49], [50]. Prenatal exposure to low doses of MXC reduces the postnatal growth [51]. Consistently, we found that the exposure to low doses of MXC perinatally decreased body weight at P0.5 and increased body weight at P45.5, thus we assumed obesity as one of the putative reasons for the differential body weights among the groups on P45.5. As IUGR has been linked to increased metabolic syndrome risk later in life [52], [53], the study of low doses of MXC exposure leading to metabolic syndrome needs to be further investigated.

Many experiments have demonstrated that estrogen or estrogenic substances can conversely inhibit steroidogenic genes, such as *Star*, *Cyp11a1* and *Cyp17a1* [54]. HPTE, a metabolite of MXC, possesses ERα agonist activity [12], [13]. In the present study, we found that *Cyp11a1* and T levels were decreased, but *Cyp19a1* and E_2_ levels were increased. Therefore, MXC could regulate *Cyp19a1* expression, and in turn leads to an increase in E_2_ production. However, how the MXC disturbs the expression of steroidogenic genes is still unknown.

The balance of androgenic and estrogenic signals plays a key role in the male reproductive system. Our lab previous results demonstrated that the mice overexpressing human aromatase possessed a multiple structural and functional alterations in the reproductive organs [41], [55], [56].

Leydig cells are a major source of T, and the total number of Leydig cells is determined during pubertal development, as their proliferative activity is limited to the prepubertal period. Estrogen inhibits Leydig cell development via ERα [57] or ERβ [38]. Hormonal regulation of the proliferative capacity of Leydig cells could be interfered by endocrine disruptors. The number and distribution of fetal Leydig cells are altered after in utero exposure to DBP or DEHP [58], [59], genestin, resveratrol, or quercetin [60]. In the current study, we observed increased number of Leydig cells at P45.5. This may be caused by the ERβ antagonist activity of HPTE [12], [13], metabolites of MXC, and/or the reduction of ERβ expression.

In this study, we demonstrated that exposure to low dose of MXC perinatally could induce the effects of decreasing or delaying of germ cell population in the testes of F1 males at onset of meiosis, as well as of downregulating the *Rarg* and Stra8, the key factors of meiosis [45], [61], [62]. The level of the meiotic marker, *Cyclin-a1*, was also reduced. Therefore, MXC was able to counteract the retinoic acid pathway, inhibit meiosis and disturb spermatogenesis.

It has been demonstrated that MXC can be metabolized into HPTE possessing antiandrogenic activity [14], T is a prerequisite for normal spermatogenesis and development [63], [64]. Here, we found that MXC decreased the serum T. These results suggest that MXC could affect spermatogenesis through the downregulation of androgen signaling pathways by antiandrogenic activity and decrease in serum T.

Taken together, the current results indicate that perinatal exposure to a low dose of MXC perturbs the testicular development in mice. This is probably caused by the multiple actions of MXC during testicular development. Firstly, MXC is able to downregulate *Cyp11a1* and upregulate *Cyp19a1*, leading to the decrease in serum T and increase in serum E_2_ levels, consequently affecting the T/E_2_ ratio balance. Secondly, the reduction of T, combined with the antiandrogenic activity of MXC metabolites, is likely to disturb spermatogenesis in P21.5 testes. Finally, the reduction of ERβ expression and/or ERβ antagonist activity of MXC metabolites may enhance the proliferation of Leydig cells in P45.5 testes. However, the exact mechanism, by which MXC disturbs the testicular steroidogenesis and spermatogenesis, is still unclear and requires further investigation. Our data provides a strong evidence of MXC effects on male mice fertility and forms the basis of a potential link between the exposure to EDCs and the increase in the incidence of male reproductive disorders for adult men.

## Supporting Information

Figure S1 **Concentrations of MXC in plasma and adipose tissue of mice treated with intraperitoneal injection or intragastrical administration of 1 mg/kg/d MXC for 5 d (n = 4/group).** Concentration of MXC was detected by ELISA. The data represent the mean ± SEM. *P<0.05.
(TIF)

Figure S2 **Representative micrographs of H&E-stained testes of mice exposed to vehicle, 0.25 µg/kg/d, 2.5 µg/kg/d or 20 µg/kg/d MXC (n = 6–8/group).** Original magnification, ×100.
(TIF)

Figure S3 **Epididymis sperm count and motility of mice exposed to vehicle or 1 mg/kg/d MXC at P45.5.** (**A**) Epididymis sperm count was determined by hemocytometer (n = 5/group). (**B**) Epididymis sperm motility was recorded using a phase contrast microscope (n = 5/group). The data represent the mean ± SEM. *P<0.05 versus vehicle.
(TIF)

Figure S4 **The related mRNA expression of** ***Esr1*** **,** ***Esr2*** **,** ***Ar*** **,** ***Nr5a1*** **and** ***Insl3*** **was normalized to** ***L19*** **level in mLTC-1 cell line cultured with DMSO or 10^−6^** **M MXC for 24 h (n = 4/group).** Vehicle-treated group was set at 1.0. The data represent the mean ± SEM. *P<0.05 versus vehicle.
(TIF)

Figure S5 **The related mRNA expression of** ***Esr1*** **,** ***Esr2*** **,** ***Ar*** **,** ***Nr5a1*** **,** ***Insl3*** **,** ***Cyclin-d2*** **,** ***Nanos3*** **,** ***Pou5f1*** **,** ***Sohlh2*** **,** ***Pcna*** **,** ***Stra8*** **,** ***Cyclin-a1*** **,** ***Dazl*** **,** ***Boll*** **and** ***Rarg*** **was normalized to** ***L19*** **level in GC-1 cell line cultured with DMSO or 10^−6 ^M MXC for 24 h (n = 4/group).** Vehicle-treated group was set at 1.0. The data represent the mean ± SEM. *P<0.05 versus vehicle.
(TIF)
