# Supplementary figures and images for: Perinatal Exposure to Low-Dose Methoxychlor Impairs Testicular Development in C57BL/6 Mice
Source: PLoS One. 2014 Jul 21;9(7):e103016. doi: 10.1371/journal.pone.0103016 (PMC4105541; doi:10.1371/journal.pone.0103016)

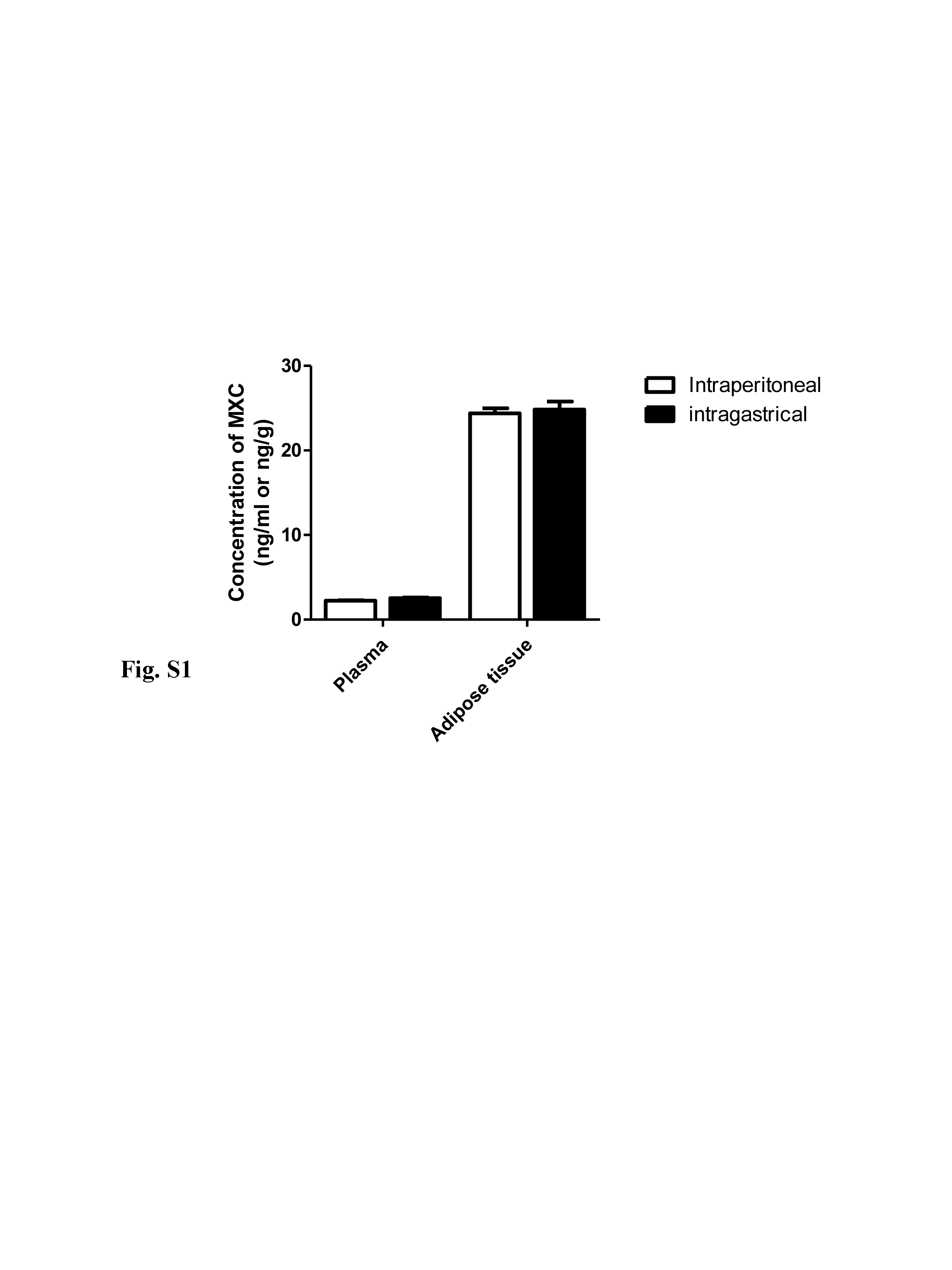

Supplement: Figure S1 — Concentrations of MXC in plasma and adipose tissue of mice treated with intraperitoneal injection or intragastrical administration of 1 mg/kg/d MXC for 5 d (n = 4/group). Concentration of MXC was detected by ELISA. The data represent the mean ± SEM. *P<0.05. (TIF) [file pone.0103016.s001.tif]

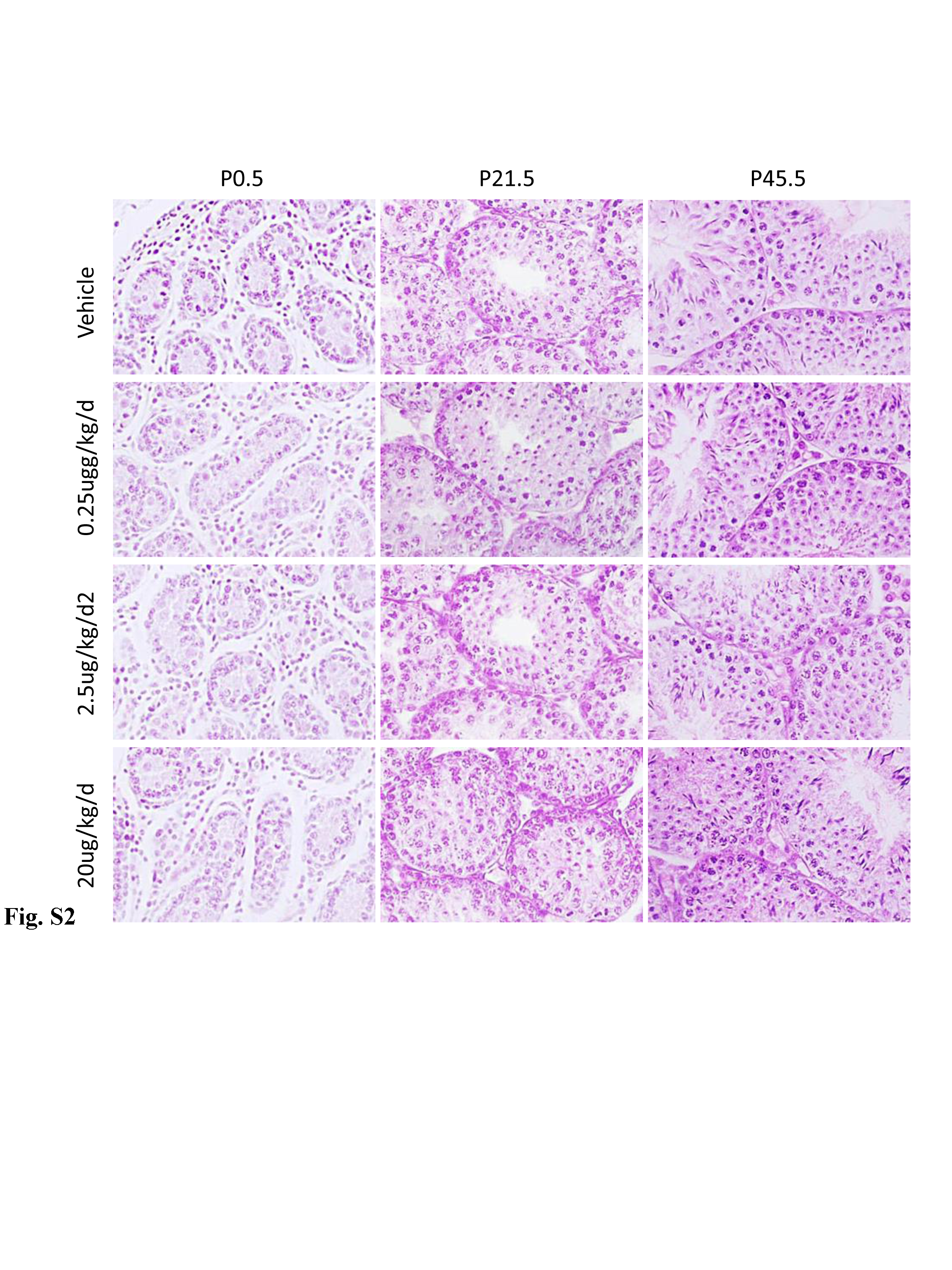

Supplement: Figure S2 — Representative micrographs of H&E-stained testes of mice exposed to vehicle, 0.25 µg/kg/d, 2.5 µg/kg/d or 20 µg/kg/d MXC (n = 6–8/group). Original magnification, ×100. (TIF) [file pone.0103016.s002.tif]

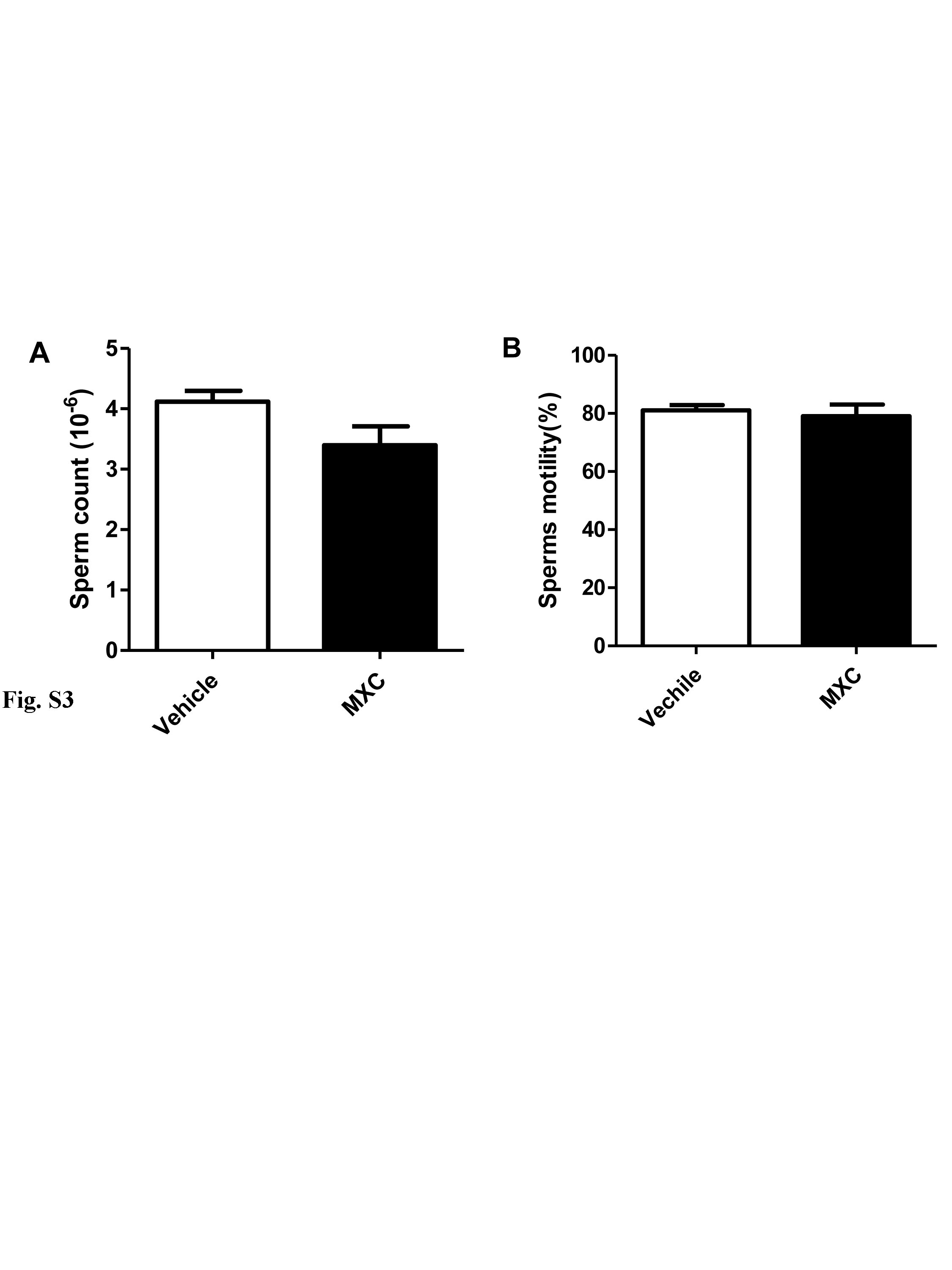

Supplement: Figure S3 — Epididymis sperm count and motility of mice exposed to vehicle or 1 mg/kg/d MXC at P45.5. (A) Epididymis sperm count was determined by hemocytometer (n = 5/group). (B) Epididymis sperm motility was recorded using a phase contrast microscope (n = 5/group). The data represent the mean ± SEM. *P<0.05 versus vehicle. (TIF) [file pone.0103016.s003.tif]

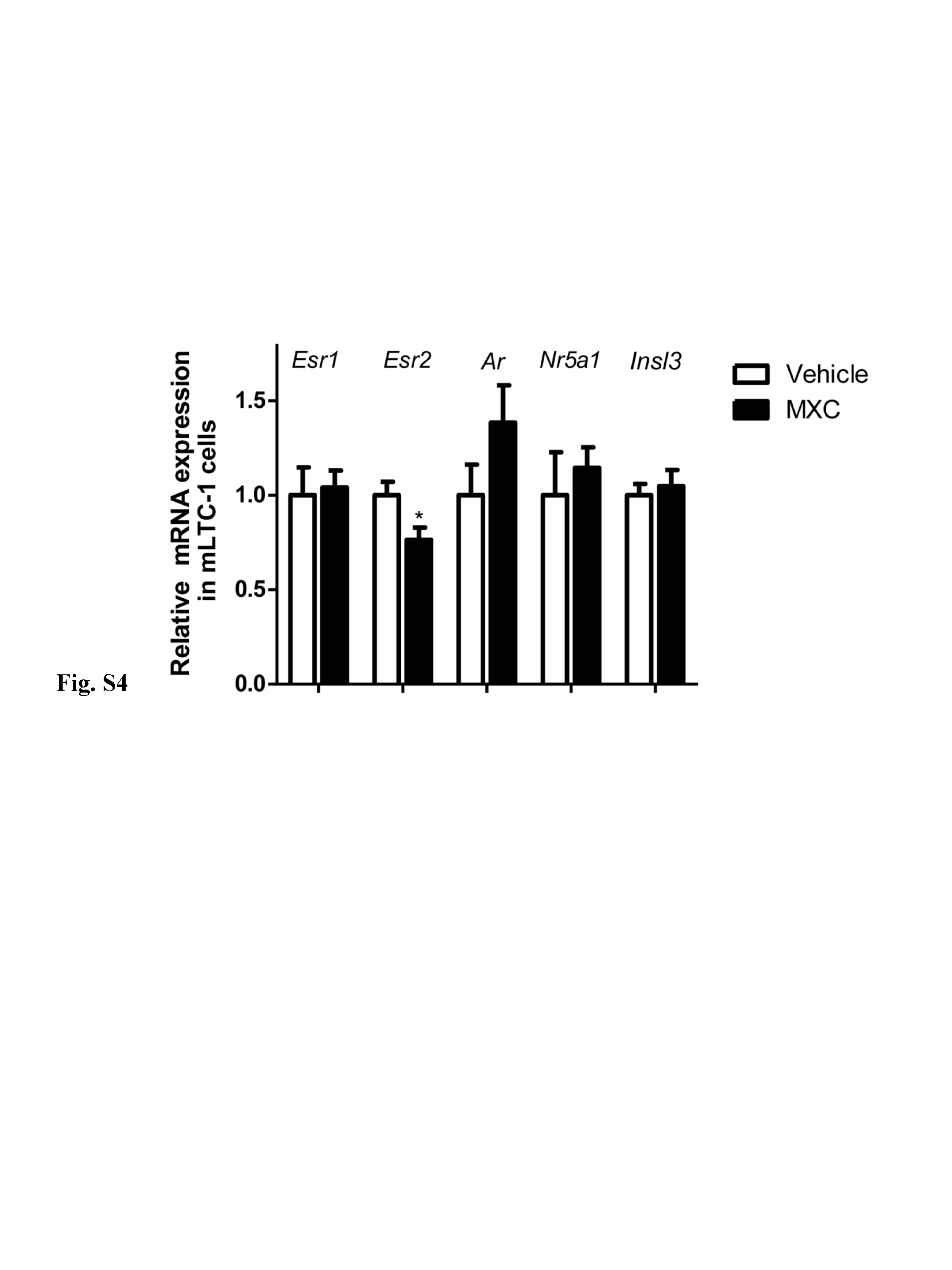

Supplement: Figure S4 — The related mRNA expression of Esr1 , Esr2 , Ar , Nr5a1 and Insl3 was normalized to L19 level in mLTC-1 cell line cultured with DMSO or 10−6 M MXC for 24 h (n = 4/group). Vehicle-treated group was set at 1.0. The data represent the mean ± SEM. *P<0.05 versus vehicle. (TIF) [file pone.0103016.s004.tif]

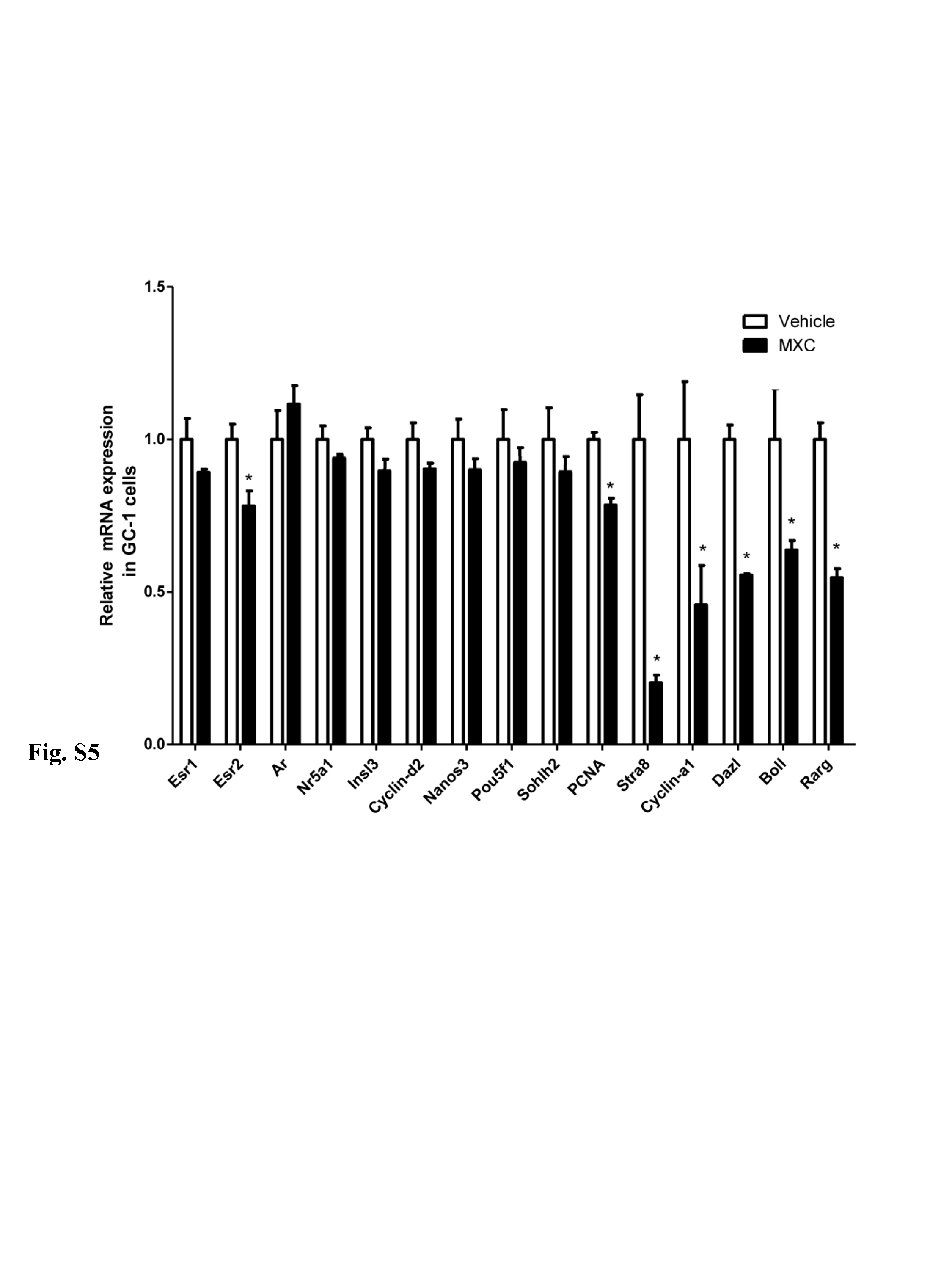

Supplement: Figure S5 — The related mRNA expression of Esr1 , Esr2 , Ar , Nr5a1 , Insl3 , Cyclin-d2 , Nanos3 , Pou5f1 , Sohlh2 , Pcna , Stra8 , Cyclin-a1 , Dazl , Boll and Rarg was normalized to L19 level in GC-1 cell line cultured with DMSO or 10−6 M MXC for 24 h (n = 4/group). Vehicle-treated group was set at 1.0. The data represent the mean ± SEM. *P<0.05 versus vehicle. (TIF) [file pone.0103016.s005.tif]
